# Born in Bradford Age of Wonder cohort: A protocol for qualitative longitudinal research

**DOI:** 10.12688/wellcomeopenres.18096.1

**Published:** 2022-10-31

**Authors:** Sufyan Abid Dogra, Kate Lightfoot, Rosslyn Kerr, Jennifer Hall, Olivia Joseph, Nasiba Siddig, Hannah Nutting, Katy A. Shire, Helen Roberts, Neil Small, Rosemary R.C. McEachan, John Wright

**Affiliations:** 1 Bradford Institute for Health Research, Bradford Teaching Hospitals NHS Foundation Trust, Bradford Royal Infirmary, Duckworth Lane, Bradford, BD9 6RJ, UK; 2 Faith in Communities, Bradford, BD3 8EJ, UK; 3 Institute of Child Health, UCL Great Ormond Street, London, WC1N 1EH, UK; 4 University of Bradford, Bradford, BD7 1DP, UK

**Keywords:** Qualitative Longitudinal Research, Born in Bradford, Age of Wonder, Health and Wellbeing, Coproduction, Cohort Study, Adolescence, Innovative Qualitative Research Methods, Growing Up

## Abstract

Born in Bradford (BiB) has followed the lives of 13,776 children born in the district between 2007 and 2011. Children in the birth cohort are now entering adolescence, and the next phase of the research - Age of Wonder (AoW) - will be a whole city cohort capturing the experiences of 30,000 adolescents progressing into young adulthood. This protocol focuses on one component of the AoW programme: qualitative longitudinal research (QLR). The study will gather in depth and detailed accounts from a sub-sample of 100 young people across four major research priorities: personal life; social and community life; growing up with difference, and growing up in Bradford. As well as using traditional qualitative methods such as interviews, focus group discussions, and ethnography, we are adopting innovative creative methods including expressions through art, activism, online and digital content, portraits, and critical events. The process of engaging in and co-producing QLR potentially provides a route to empowering young people to shape the narrative of their own lives as well as informing intervention development.

## Introduction

### Background of Born in Bradford birth cohort

The Born in Bradford (BiB) study was established in 2007 to examine how genetic, nutritional, environmental, behavioural and social factors affect health and development during childhood. Between 2007–2011 the study recruited 12,453 pregnant women who experienced 13,776 pregnancies and included 3,448 partners. Forty-five percent of mothers in the cohort are of Pakistani origin and over half live within the fifth most deprived areas of England and Wales. The BiB cohort was established with broad aims to: describe health and ill health within a multi-ethnic (largely bi-ethnic), economically deprived population; develop, design and evaluate interventions to promote health; provide a model to support evidence-based practice within the National Health Service (NHS) and other health-related systems; and build and strengthen local research capacity in Bradford. Protocols for the original study, follow up studies, and cohort descriptions have been published ( Bird *et al*., 2019; Wright *et al*., 2013).

The BiB team advocates Bradford as a ‘City of Research’, supporting the campaign with Bradford Institute for Health Research to develop ‘citizen science’ and encourage all who live in the Bradford District to contribute to health research. Over 300 papers using BiB data have been published on a range of topic areas including environmental influences on health such as air quality ( Pedersen *et al*., 2013) and green space ( McEachan *et al*., 2018); genetic factors, e.g. gene knock-outs ( Narasimhan *et al*., 2016); congenital anomalies ( Sheridan *et al*., 2013); fine-scale population structure ( Arciero *et al*., 2021); mental health ( Prady *et al*., 2016); obesity ( Wright *et al*., 2016); physical activity and sedentary behaviour ( Collings *et al*., 2020a; Collings *et al*., 2020b; Hall *et al*., 2021); using religious/cultural settings for childhood obesity prevention ( Dogra *et al*., 2021; Rai *et al*., 2019); education ( Pettinger *et al*., 2020); and COVID-19 ( Bingham *et al*., 2021; McEachan *et al*., 2020; Pybus *et al*., 2022). A summary of key findings can be found at https://borninbradford.nhs.uk/our-findings/.

### Age of Wonder: BiB research plans for the next seven years

**Figure 1.**
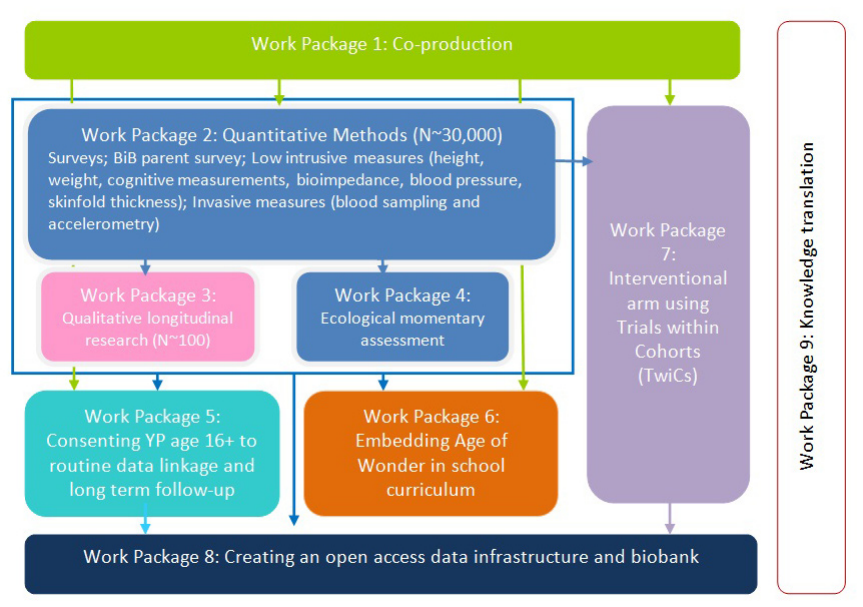
AoW work packages for all components of full research programme.

Age of Wonder (AoW) is the next stage in the development of BiB’s cohort research focussing on adolescence and young adulthood. Between 2022 and 2029, we will work with around 30,000 young people in Bradford using a range of approaches, including questionnaires, health measures, motor and cognitive measures, and qualitative longitudinal research (QLR). The QLR component will follow 100 young people through their journeys to young adulthood, from 12/13 to 19/20, providing insights into young people’s experiences of growing up in Bradford. As well as the qualitative standalone value, we expect to synthesise this work with AoW quantitative data to provide a rich understanding of this transformative phase of life (see Figure 1).

### Qualitative longitudinal research background

QLR holds the potential to investigate and describe complex relationships around continuity and change ( Lloyd *et al*., 2017), grasp subjective meanings ( Heinz & Kruger, 2001), measure change and associated processes ( Farrall, 2006; Hanna & Lau-Clayton, 2012), and complement evaluation of programmes that use different methods ( Barnes *et al*., 2005; Corden & Miller, 2007; Irwin, 2011). Moreover, Denzin, Lincoln & Giardina (2006, p.776) note the value for scientists in maintaining ‘collaborative, reciprocal, trusting, mutually accountable relationships with those we study’, in creating robust qualitative research to understand constructions of ‘truth’, worldviews, and interpretations of events shaped within particular social environments, at particular points in time. 

Previous studies undertaking QLR with adolescents have highlighted the importance of a wide repertoire of approaches to gather data ( Henderson *et al*., 2006; Neale & Bagnoli, 2007; Neale, 2020). Thomson & Holland (2003), for example, acknowledged that it is not ‘normal’ for young people to be invited by researchers to take part in standardised interviewing repeatedly. To avoid this limitation, our QLR with young people employs various data collection techniques such as focus groups ( Bagnoli & Clark, 2010) and arts-based approaches ( Bagnoli, 2009; Daykin, 2020).

AoW’s QLR will collect an in-depth record of young people’s changing contexts, attitudes and experiences through adolescence. This exploration of their lives over time will not only give insights into the ways in which their identities and relationships are negotiated, but also related factors that may impinge – positively or negatively - on their health and wellbeing ( Morrow & Crivello, 2015). Such insights can be examined alongside the ways in which young people engage with external influences (family, peers, neighbourhood, school, clubs, leisure, wider society), allowing consideration of the implications of these for social policy ( Treanor *et al*., 2021). Combining social contexts (e.g. political, social, and economic) and personal insights creates ‘thick data’ ( Geertz, 2008; Wang, 2013), which can contribute to interventions designed to improve education, health and wellbeing, and inform social policy that impacts on young people ( Corden & Miller, 2007).

### Aims of qualitative longitudinal research

Our overall aim is to establish a sustainable qualitative longitudinal study that complements other work packages in a cyclical way. This will be achieved through detailed longitudinal inquiries and co-production with young people on how best to capture details of their lives. We will use a range of data collection methods to provide a voice for young people, contributing both to the richness of AoW data and to QLR methodological development. Our QLR workstream within AoW provides a route for, and offers support to, young people to input into programme design, operation, intervention development and dissemination of data. Further aims are to:

1. Develop a methodologically innovative, robust, QLR workstream of AoW with a focus on seeking insights into the internal, social and community lives of young people.
2. Make qualitative data available in a way that can connect with quantitative AoW data, and data from other national projects to help explore the impact of societal changes, for example shifts in economic and social policy.
3. Triangulate, interrogate and draw on the AoW quantitative dataset to enrich all parts of the study.
4. Undertake focused qualitative studies to investigate specific research questions.
5. Provide support for intervention development and evaluation for other AoW workstreams and facilitate them in expanding qualitative components of their work.
6. Harness young people’s insights to adapt and prioritise social, epidemiological and biomedical research in the broader AoW research programme.
7. Support young citizen scientists in developing ways of investigating, recording, and sharing insights into their own lives, facilitated by qualitative research methods.

## Methods

### Sampling

Our sample will comprise 100 young people aged 12–13 years at recruitment . They will be recruited from schools (including alternative provision), community and faith settings, sports and arts venues, and through BiB’s existing network of parents and families, using purposive sampling.

They will be recruited in three tranches, 34 in year one, 33 in year two and 33 in year three. ^1^ Of these, around two thirds will be invited to participate on the basis of demographic characteristics (gender; ethnicity; socio-economic context; geographical location; family circumstances), which broadly reflects the overall distribution in the wider BiB cohort. The remainder will be invited as members of groups often under-represented in genuinely participative co-production, including for instance children in special education, looked after children, and children of refugees ( Roberts *et al*., 2018). Recruitment in tranches will allow us to consider differences in young people’s experiences of the same age in different years ( Morrow & Crivello, 2015) and is likely to include issues such as the impact of the COVID-19 pandemic on education, mental health and cost of living. More practically, recruitment in tranches will facilitate the administration of cohort recruitment in a study where all recruits will be supported to stay in the cohort throughout the seven years from recruitment. We will encourage participants to remain a part of our QLR even if they move away from Bradford, in which case we will facilitate data collection regardless of their location via online methods including Zoom and Microsoft Teams.

Minimising attrition involves building quality research relationships with participants through repeat engagement, continuing conversations and sound record keeping compliant with both GDPR and best practice safeguarding ( Denzin *et al*., 2006; Teague *et al*., 2018; Treanor *et al*., 2021).

### Consent

Our QLR study will seek opt-in consent. Consent in studies of this kind is a process and not an event ( Guillemin & Gillam, 2004; Moore *et al*., 2018; Rooney, 2015; Warin, 2011). Consent will be viewed and applied as a fluid, situational, repeated, and ongoing process.

At initial recruitment, we will seek parental written informed consent and assent from young people (who will be aged 12/13 years). In ensuring consent which is well-informed, ethics documents will be designed for the needs of participants (and parents). This will include translations for parents or newly arrived young people who do not speak English, dyslexia-friendly fonts, Makaton and alt-text on images. ^2^ Parents and young people will be provided with information sheets detailing initial data collection activities including re-consents as the study progresses since practices for obtaining informed consent will be informed both by ‘Gillick competence’ and co-production ( NSPCC Learning, 2020). If a researcher has concerns about the Gillick competence of a young person they will work with the young person, teachers and parents to determine whether a model of parental consent and young person assent is more appropriate. Health Research Authority guidance ( NHS Health Research Authority, 2021) helpfully reminds researchers that:

*“A child / young person's right to give consent is dependent upon their capacity to understand the specific circumstances and details of the research being proposed, which in turn will relate to the complexity of the research itself. Children and young people's competence may well be reflected in their ability or otherwise to understand and assess risk. Competence to understand will be heavily influenced by how the information is presented to the child or young person, and the language used. You must ensure that you maximise a child / young person's chances of understanding what is involved in your study.”*

We will be working on mechanisms and processes to minimise attrition, while enabling and empowering young people to withdraw should they wish to do so - particularly being mindful of power dynamics which can be involved with parental consent and young people’s assent ( Liabo *et al*., 2017; McEvoy *et al*., 2017). Alongside our study, we will run a variety of in-person and online workshops so that participants and parents can fully understand what being in a research project means, their right to withdraw at any stage, and the ways in which they can signal if they would like to withdraw from any parts of a discussion.

### Coproduction, involvement and engagement of young people

QLR research priority areas were co-produced with a small, diverse group of young people from Bradford but not from the QLR sub-sample, during AoW and QLR research programme design prior to the submission of the funding application to Wellcome. These young people were recruited from BiB’s existing links with groups of young people in schools, community organisations, mental health and youth services in Bradford. Between February 2021 to September 2021, participants attended a series of group discussions and workshops in which they were invited to reflect on their lived experiences and define research priorities that they would like the Age of Wonder research project to explore further.

The themes in Table 1 represent the outcomes of initial co-production with young people. A defining feature of our QLR is ongoing co-production and co-design of qualitative methods of social inquiry, meaning that these research priority areas, and the methods discussed below, may evolve as we continue co-production with young people over the next seven years. To this extent, a QLR/co-production protocol is very different (and somewhat less detailed) than a protocol for, for instance, a randomised controlled trial.

**Table 1.** Research priority areas for Age of Wonder qualitative longitudinal research.

Research priority area	Related topics as identified by young people in initial co-production
Personal life	Identity – ‘who you are’, how you ‘fit’ with your peer group Expectations of future and fears of the unknown Puberty and sexual encounters (from age 16) Social media – ‘gone viral’ and the part it plays Body image – comparing self and others Hopes, aspirations and ambiguities (individual and community) Health and wellbeing - staying healthy Positive and negative influences on life Art and culture
Social and community life	Family relations and home environment School and the community around you Role models and power of positive influence Culture shaping growing up
Growing up with difference	Positive and adverse experiences; discrimination, poverty and surroundings, life in council estates and deprived neighbourhoods
Growing up in Bradford	Multi-ethnic encounters and growing up in diverse Bradford; racism Criminality, drugs, anti-social behaviour Living with faith or no faith; ideals of purity, spirituality and devotion in Bradford Economic practices/choices in Bradford: transitioning into workforce Environment, ecology and sustainability

The topics in Table 1 above, identified from early co-production, will not be used to limit the focus of research. Within an approach that is sufficiently structured to provide a framework, we will be guided by regular, open conversations with young people in which they will be encouraged to talk about the experiences that shape their journey through adolescence. As well as our core study participants, the wider work of AoW with community groups, schools, religious settings, sports organisations, mental and sexual health services, educational institutions and the families will enable wider insights as we develop our QLR. ^3^ By collaborating with local place-based groups and community organisations, QLR will explore and expand the presence of young people in research, data collection, and programme implementation.

The open nature of conversations as part of the co-production of QLR will, we hope, ensure that young people feel part of the longitudinal process and the AoW community as young citizen scientists. We will be testing and observing whether the sense of enjoyment and fun we hope to engender will ensure strong retention and minimal attrition over the seven-year duration of the project.

### Qualitative longitudinal research

In recent years, QLR has seen a period of methodological innovation ( Denzin & Giardina, 2006; Neale, 2021; Onwuegbuzie *et al*., 2010) building on social media analysis, artistic expression, youthful activism, and critical events in young lives. We propose to build on these methods in ways that resonate with young lives in the 2020s ( Denzin *et al*., 2006). Alongside traditional methods of QLR, such as interviews and focus groups, over the next seven years our QLR will employ a range of techniques that equip young people to study and report on their own lives, develop the critical skills that foster citizen scientists, and allow young people to identify pathways which they may not previously have considered.

Table 2 with qualitative data collection practices in the left-hand column is what we aspire to achieve, however the granularity of methods will be shaped by co-production with young people and discussions of ethics. The numbers in the right-hand column indicate the number of participants (from the sample of 100) that will be followed longitudinally over seven years. Every young person in the sample of 100 will be followed longitudinally for the duration of the study, but young participants will have differing levels of involvement in the data collection methods and practices. For example, all young people will be invited to submit a short expression of their hopes and fears for the future on an annual basis, whereas 40 will participate in in-depth interviews, and 25 will take part in portrait sessions with our artist in residence.

**Table 2.** Qualitative longitudinal research methods. Planned data collection over study duration (7 years per participant).

Qualitative data collection methods and practices (repeated annually)	Sample size
**Short expression:** Insights on how young people change their future aspirations, plans and ambitions over time. This will include an annual short expression – written, spoken, filmed or pictorial from young people.	N = 100 (Y1 = 34; Y2 = 67; Y3 = 100)
	Total over study duration: 700
**Group discussions (in groups of 8):** To acquire information from diverse group of young people on a specific theme	N = 80 (Y1 = 27; Y2 = 54; Y3 = 80)
	Total over study duration: 560
**Online/ digital content:** To gather information through content analysis on everyday life and priorities of young people through online/digital sources like social media like TikTok, Instagram, BeReal, YouTube, Facebook, WhatsApp etc.	N = 50 (Y1 = 17; Y2 = 33; Y3 = 50)
	Total over study duration: 350
**In-depth interviews:** To explore in detail thoughts and feelings related to priority topics	N = 40 (Y1 = 14; Y2 = 27; Y3 = 40)
	Total over study duration: 280
**Expressions through art, culture and youthful activism:** To gain insights and familiarise with the imaginative worldviews of young people by collecting and analysing the artistic expressions produced, created and lived by them like drawing/sketches, poems, performances, music, environmental and political activism etc.	N = 25 (Y1 = 9; Y2 = 17; Y3 = 25)
	Total over study duration: 175
**Portraits:** To document young people's hopes and fears for the future through an annual interview and portrait session with a professional photographer.	N = 25 (Y1 = 9; Y2 = 17; Y3 = 25)
	Total over study duration: 175
**Critical events:** To gather in-depth understanding of everlasting impressions left on young people’s lives through critical events like entering into new school, changing family circumstances, pleasant or unpleasant peer experience, enhanced interest in particular sports, changes in food habits, puberty and sexuality, trauma, fight, drugs, poverty/unemployment or other life defining events like grief or happiness	N = 5 (Y1 = 1; Y2 = 3; Y3 = 5)
	Total over study duration: 35
**Life history:** To collect a cumulative record of changing perceptions and attitudes amongst young people on health and wellbeing and lived experiences with the outside world throughout adolescence, by drawing on all QLR methods of data collection, and secondary data.	N = 10 (Y1 = 2; Y2 = 6; Y3 = 10)
	Total over study duration: 10
**Ethnography:** To gather insights of social life of young people through participant and nonparticipant observation by taking part in various social gatherings of young people, and triangulation of data collected from various sources about their everyday lives.	N = 10 (Y1 = 2; Y2 = 6; Y3 = 10)
	Total over study duration: 10
**WhatsApp Group:** Young people create, and administrate WhatsApp groups independent of adults and researchers. This is a citizen science data collection activity where young people inform researchers about relevant topics and updates within WhatsApp groups as they see fit.	Optional for all 100 participants

As 34 young people will be recruited in year 1, an additional 33 in year 2, and the final 33 in year 3 to make up the sample of 100, the numbers in brackets indicate the proportional sample size for data collection from each method. Each recruit will complete a cycle of longitudinal study, meaning that66 out of the 100 young people selected in years 2 and 3 will continue as part of QLR for a further 12 or 24 months in order to complete the overall study duration of seven years.

The rationale behind the inclusion of the methods in Table 2 is to generate a comprehensive understanding of the multiplicity of factors influencing how young people in Bradford live their lives, and how this may change over time. There are limitations to relying solely on traditional qualitative methods, as no data, however collected, can reflect every aspect of all individual experiences. The inclusion of innovative methods will allow us to better connect with the diversity of young people’s lived experiences, as each method highlights a different aspect of young lives in Bradford, ‘reaching the parts that other methods cannot reach’ ( Pope & Mays, 1995, p.42). Moreover, following all young people in the sample for the duration of the study across a range of data collection methods will generate insights into the factors that shape their experiences of growing up.

### Data analysis

Given the breadth of data our QLR seeks to collect, a range of analytical and theoretical approaches will be used. Much of the data will be analysed thematically using a framework analysis ( Gale *et al*., 2013) combined with integrative analysis of longitudinal qualitative data. While thematic analysis ( Braun & Clarke, 2006) is likely to be the most useful tool for the data collected through in-depth interviews and group discussions, content analysis ( Hsieh & Shannon, 2005) is likely to be more useful for short expressions and online digital content. Discourse analysis ( Cheek, 2004) will explore how young people describe critical events and how their perception changes as they grow up.

### Reflexivity

Mitchell *et al*. (2018, p.673) posit the importance of reflexive practice in qualitative research. Researchers in this study will be expected to reflect on, and describe, the ‘contextual intersecting relationships (e.g., race, socio-economic status, age, cultural background) between the participants and themselves’ ( Berger, 2015; Dodgson, 2019, p. 220) and whether they have shared experiences with study participants ( Dodgson, 2019). The researchers in our QLR are ‘outsiders’ (all being adults), so reflecting on power differentials will be important ( Grove, 2017; Mathijssen *et al*., 2021). Reflexivity will be an inbuilt feature of key operational meetings prior to, during and after data collection, analysis and dissemination ( Onwuegbuzie *et al*., 2008). Such reflexive moments will allow consideration of ethically important moments, particularly during data collection ( Guillemin & Gillam, 2004).

Power relations and peer relations can never be fully mitigated in these situations. However, by ensuring that our initial results presentations go to the young people involved prior to wider publication and dissemination, and differences in interpretation are fully discussed and reported alongside those in our own discussions, we expect to present a rounded picture.

### Dissemination and engagement

As a community-focused project, AoW will complement traditional academic dissemination with a variety of community-focused dissemination and engagement strategies flexibly informed by young people. Some of this will be dictated by capacity and resources, including wider media dissemination. The list below details the planned research dissemination and engagement activities for the duration of the study. Because co-production is a priority for our QLR, the list below is not exhaustive, as there is scope to develop dissemination and engagement techniques identified by participants ( Research Retold, 2019).

- **Yearly exhibition:** Showcase of annual short expressions on changing perceptions of young people about their hopes, aspirations and fears to be displayed in the city.
- **Newsletter:** Annual newsletter to young people and families detailing young people's participation.
- **Stakeholder presentations:** Presentations of young people's participation and project milestones to stakeholders such as schools and community organisations.
- **Performing arts:** Working with young people and local drama groups to develop various forms of performance art based on young people's experiences.
- **Board game or app:** During early adolescence, participants will contribute to the creation of a 'Snakes and Ladders' style board game to highlight key challenges and facilitators of a positive experience of growing up in Bradford. During late adolescence/early adulthood, participants will contribute to the design of a smartphone app related to growing up in Bradford.
- **Portraits:** Alongside excerpts of discussion with photographer on hopes and dreams, portraits of young people showcased on various platforms
- **Art:** Working with stakeholders to produce art such as illustrations, comics, and short animations, which highlight the project's key findings.
- **Sports/fun days:** Organising sports/fun days to promote physical activity and access to greenspaces for young people
- **Girls’ knowledge café:** Bringing young girls together in communities to share their experiences in a safe space. Girls can rotate amongst groups and hear from role models from the local community
- **Social media:** Sharing project progress and findings through social media posts (e.g. Instagram; YouTube; Twitter; Facebook; TikTok).
- **Mainstream media:** Dissemination of findings via mainstream media (e.g. television, radio, newspapers, magazines, podcasts).
- **Academic dissemination:** Sharing findings via traditional academic channels, such as publications and conference presentations. These research outputs will be accompanied by short sound bites, providing a summary of relevant information.

### Transparency and use of data

Transparency around the use of QLR data is a key ethical consideration in dissemination and engagement. By virtue of the range of dissemination techniques outlined above, it will be possible to involve young people in certain aspects of data analysis and identifying key information for dissemination. Consent to use young people’s data in dissemination and engagement will be collected at the same time as consent to participate in data collection. All data will be anonymised before dissemination - unless young people choose to be identified - apart from portraits, where participants’ identities will be clear.

Participants and their parents/carers will not have to re-consent each time their data is used. Throughout their participation in QLR, young people will be reminded of their right to withdraw - both their participation, and their data. If a participant withdraws, they can indicate that they do not wish their data to be used in further analyses and publications. Similarly, once data are collected, participants will not have the opportunity to rewrite them at a later stage. However, throughout the study, participants will have the opportunity to reflect on their previous contributions. This decision is based on preserving authenticity and reducing social desirability bias.

### Ethical approval

Ethical approval for QLR is part of the overall AoW project and has been granted by NHS Leeds Bradford Research Ethics committee (Approval number ref: 21/YH/0261, date 22.12.21). Regulatory approval for AoW has been granted by the Health Research Authority. Initial ethical approval was granted for QLR to recruit young people to take part in QLR with yearly submission of young people’s ‘short expressions of hope’ with consent from parents to request this every year for the next seven years from their child. Initial ethical approval has also included approval for all of the broad data collection methods. As the specific research focus changes and develops to cover the different areas identified by young people through co-production, ethics amendments will be submitted to gain approval for any new research materials (e.g., interview schedules or focus group topic guides) or additional research required to fully explore the topic.

Whilst we expect there to be no risks greater than the mundane risks of everyday life to participants of taking part in the Age of Wonder study, no activity is entirely risk free. It is possible that the planned data collection techniques, particularly interviews, may uncover potentially sensitive or upsetting topics for participants. It is also possible that research activities will reveal safeguarding concerns for the participant or their family. It is made clear in participant information sheets and consent forms that a researcher may break confidentiality if they are concerned about the safety of a young person or their family. ^4^ In such cases, the researcher will follow the safeguarding policy of Bradford Teaching Hospitals NHS Foundation Trust and report the safeguarding disclosure. It is likely that during data collection, lone working within community locations (e.g. community centres, public spaces, and participants’ homes) will be required by QLR research staff, in which case researchers will follow the Bradford Teaching Hospitals NHS Foundation Trust policy for working alone to protect as far as is reasonably possible the safety of researchers as well as participants. This policy includes provisions such as a nominated person for contact and further action if communication from the researcher to confirm their safety within set timescales is not reached.

## Study status

Participant recruitment is due to begin in September 2022 (the start of the 2022/23 academic year), and data collection will follow shortly thereafter.

## Discussion

This protocol has outlined the potential of QLR to understand the multiplicity of young people’s lived experiences of growing up in Bradford. The defining features of our approach are: innovative data collection methods; the synthesis of thick data and big data (findings from a large quantitative dataset, N = ~30,000); making participation fun, and the exploration of mapping data on political, social, and economic change onto QLR data.

A central feature of our QLR is the multiple data collection methods repeated with the sample. These extend beyond the ‘traditional’ qualitative interview, focus group, and ethnographic observation used in much qualitative research to build a detailed and holistic understanding of the lives of young people. Using a range of methods will reveal the ways in which young people construct and understand their life worlds and the role they have in the social system of which they are a part ( Denzin *et al*., 2006). The novel methods will be used to collect data and make qualitative observations through artistic expressions, critical events in life, youth activism, social media and portraits. Our QLR will empower young ‘citizen scientists’ to actively inform us on data collection methods by decolonising, democratising, and decentralising the ways in which qualitative data is traditionally collected (in-depth interviews and focus group discussions).

Moving away from the reliance on stand-alone qualitative data, our QLR will juxtapose qualitative and quantitative findings, whilst also exploring additional methods of synthesis ( Popay *et al*., 2006). AoW’s large quantitative data set will not only inform qualitative inquiries, but, by benefiting from thick data, will be reciprocal and cyclical to inform the design of large questionnaires that AoW will use to gather big data. Synthesising data in this way seeks to avoid the ‘add-on’ status of qualitative research sometimes found in mixed methods health research ( O’Cathain *et al*., 2014, p.121), and is essential in understanding complex phenomena including young people’s lived experiences of growing up in Bradford.

We acknowledge the limitations of qualitative data collection through institutions. Hence, our QLR will step away from total reliance on institutions for data collection with young people. We believe chat groups, social media, sports teams, street/community-based networks of young people, active participation in religious gatherings, youthful activism, and civic participation in art, culture, poetry, music and other community voluntary events can be the sites where we will observe young people and collect data. These new sites function as entertaining venues of data collection for young people highlighting young voices and lives. We advocate that data collection from young people can be fun as well as what young people may describe as entertainment can be the sites of data collection.

Another key feature of BiB’s research is co-production and involvement of local communities ( Islam *et al*., 2022). The co-production of our QLR, and overall AoW, with young people will, we hope, demonstrate a range of practical as well as methodological and intellectual benefits and outcomes ( Roberts *et al*., 1994) – but this is not a given. Longer term follow-up shows negatives as well as positives can be observed ( Roberts *et al*., 2011). We remain aspirational and optimistic that co-production can foreground the insights of young people in seeking to understand their changing lives in the AoW programme. Triangulation of research findings across diverse and novel qualitative methods, including QLR, will allow us to work on the co-production of interventions to improve health, educational outcomes and wellbeing. For young people, we believe that their involvement in this work will enhance their awareness of what the current evidence base is for interventions and tell us how these might be improved or transformed. Are we addressing their priorities as well as ours? Furthermore, co-production of QLR with young people will mean that they can become citizen scientists by participating in community and place based research initiatives where they can be equipped to work on and evaluate measures that may impact on their lives and communities. Providing a route to engage young people in describing and shaping a narrative of their own lives can be emancipatory.

Given that political, religious and economic structures and events directly shape young lives, we will map data on political, social and economic change onto our QLR data to see whether our own data map the impact of recession, the cost of living crisis, welfare reform and what comes next in the pandemic world on young people’s lives. At the same time, we will be recording views of young people on changing political and social landscapes, family relations, local elections and inter-cultural, inter-ethnic and cross-neighbourhood patterns of interaction. Our QLR is an attempt to describe and understand how young lives are lived in modern Britain and how these young people describe their experience of growing up as different to that of their parents and siblings.

## Data availability

### Underlying data

No data is associated with this article.

## Footnotes

^1^ On the completion of seven years, 66 out of the 100 young people selected in years 2 and 3 will continue as part of QLR for a further 12 or 24 months in order for 7 years of follow up for all. Funding sources for this period are currently being appraised for early funding submissions, which will include early findings from young people recruited in year 1.

^2^ Alt-text helps screen-reading tools describe images to visually impaired readers.

^3^ Please see https://www.bradfordhospitals.nhs.uk/born-in-bradford-age-of-wonder-project/ and https://borninbradford.nhs.uk/what-we-do/schools/age-of-wonder/ for an overview of the Age of Wonder project.

^4^ These ethics documents will also include signposting to services able to give support for mental health, domestic violence, child abuse and education needs.
