# Supplementary material for: Born in Bradford Age of Wonder cohort: A protocol for qualitative longitudinal research
Source: Wellcome Open Res. 2023 Oct 10;7:270. Originally published 2022 Oct 31. [Version 3] doi: 10.12688/wellcomeopenres.18096.3 (PMC10362373; doi:10.12688/wellcomeopenres.18096.3)
Supplement: Peer review history

# Reviewer report: Reviewer response for version 2

**DOI:** 10.21956/wellcomeopenres.21240.r62310

**Published:** 2023-07-21

**Authors:** JD Carpentieri

**Affiliations:** 1 University College London, London, England, UK

**General comments**

- I’m thrilled to see the development of the Age of Wonder cohort, and even more thrilled to see that you are integrating qualitative longitudinal research into this cohort from the start. While the UK is a global leader in cohort study research, the country has not done nearly as well at integrating qualitative research into its major cohorts. I’m very glad to see BiB bucking that trend through this ambitious and important project. 
- It was interesting to read reviewer 1’s thoughtful comments and the authors’ equally thoughtful responses.

**Abstract**

- It would be helpful for the abstract to provide a bit of information about Bradford. At a minimum I’d suggest mentioning that it is in the UK: this isn’t something that international readers would necessarily know. But ideally you’d also say a little bit more to indicate why Bradford is a particularly interesting site of study. I suspect that including this information in the abstract will help you capture more readers’ interest.

**Introduction**

- Good introduction. The sentence “Forty-five percent of mothers in the cohort are of Pakistani origin and over half live within the fifth most deprived areas of England and Wales” is potentially confusing though. By “fifth” do you mean “one-fifth” i.e. the bottom quintile or do you literally mean the number 5 most deprived area in a ranked list of all UK areas? Logic dictates that it’s the former, but the wording is slightly unclear.
- When discussing previous studies undertaking QLR with adolescents, you cite some valuable UK research but may also want to consider international QLR research on adolescence, in particular the mixed methods Young Lives project. There is much that can be learned (empirically and methodologically) from the Young Lives team’s work in countries such as Ethiopia.
- In your “Aims” subsection, I was interested to see that you plan to “undertake focused qualitative studies to investigate specific research questions”. Are you able to say how these focused studies fit in with the plans outlined in Table 2? E.g. do you plan on using some of the different approaches outlined in Table 2 to undertake these focused studies?

**Methods**

- I like your “recruitment in tranches” approach. In addition to the benefits you cite, this recruitment strategy will also enable you as a research team to be a bit more reflective and strategic about recruitment then you might have been if you are recruiting 100 participants in one go. It would also reduce the likelihood of being overwhelmed in Year 1 of data collection.
- I’m glad that you will continue to follow individuals who move out of Bradford, and agree with your list of benefits to this approach. There may for example be interesting findings when comparing young people who choose to live at home while attending university versus those who move away for university.
- On the notion of consent, universities’ ethics processes often err on the side of bombarding potential participants with information, so that universities are protected but participants are no wiser. I’m thinking for example of overly wordy information sheets that cause participants to think “this is too long to read so I’ll just sign it”. I’m glad to see that you are going to be taking a richer and more holistic approach to informed consent. This is particularly important with regard to issues such as reading level (as mentioned by Reviewer 1).
- In Table 1, “Art and culture” appears under “Personal life”. I’m not saying this is the wrong place, but art and culture definitely stands out as somewhat different from more obviously personal issues such as body image. For the category “Social media” you have provided a bit of extra text to suggest how this category might fit into one’s personal life. It might be useful to do the same for “Art and culture”.
- I applaud the “citizen scientists” aspect of your project. However you don’t really explain what you mean by this term until the penultimate paragraph of the paper. It would be helpful to bring this explanation forward.
- Can you say what you mean by “allow young people to identify pathways which they may not previously have considered”? If this means that you expect your research to potentially shape rather than merely capture your participants’ lives, it would be useful to reflect on this. Such reflections would be very interesting, particularly given the fact that the vast majority of cohort study research posits itself as being as neutral as possible, with regard to influencing study members’ lives (with obvious exceptions such as the discovery of threatening health conditions).
- In Table 2, I think your “different Ns for different methods” approach is sensible. If you chose to use all types of approaches on all 100 participants, your project would likely become almost exclusively a data collection and management exercise, rather than an analytic study. (There are some excellent Timescapes-related articles on this phenomenon.) My only real question here is with regard to the “Critical events” category: 5 seems like a small number. Would it be possible to push this number up to 8-10 in order to capture a wider set of experiences of critical events? I’d also be tempted to push the life history number up to 12-15 if possible. But you have to make decisions based on your resources – and in my experience it’s important to be brutally realistic about what can be accomplished in a large qualitative longitudinal study.
- You suggest a broad range of data analysis strategies. I wonder if narrative analysis might also be useful – this is a particularly valuable approach for exploring identity development over time. It might also be useful for exploring some of the important issues discussed in paragraph 2 of your Discussion.
- In the “Transparency and use of data” section I would expect to see information about what the research team will eventually do with the collected data. For example, ESRC guidelines require UK birth cohort data to be deposited with the UK Data Service so that other researchers can use the data, unless it is impossible up for such data to be sufficiently de-identified. Will your data eventually be made available to the broader research community? If not, it would be helpful to discuss the reasons for keeping it proprietary.
- Under “Study status” you refer to September 22 in the future tense. Given the evolving nature of this article, you may want to tweak that.

**Discussion**

- You sensibly suggest that your study will “juxtapose qualitative and quantitative findings, whilst also exploring additional methods of synthesis”. Given the richness of the qualitative and quantitative data that will be available to you, you may want to explore the production of mixed methods pen portraits such as those outlined in Carpentieri, Carter & Jeppesen (2023) ^1^. Laura Sheard has of course done interesting work on qualitative pen portraits.
- I’m pleased to see that you want to make the process fun/enjoyable for participants. Your coproduction strategies will be absolutely essential to achieving that ambition. 

Is the study design appropriate for the research question?

Yes

Is the rationale for, and objectives of, the study clearly described?

Yes

Are sufficient details of the methods provided to allow replication by others?

Yes

Are the datasets clearly presented in a useable and accessible format?

Not applicable

Reviewer Expertise:

Qualitative longitudinal research; Cohort studies

I confirm that I have read this submission and believe that I have an appropriate level of expertise to confirm that it is of an acceptable scientific standard.

## References

[1] : Between life course research and social history: new approaches to qualitative data in the British birth cohort studies. *International Journal of Social Research Methodology* .2023; 10.1080/13645579.2023.2218234 1-28 10.1080/13645579.2023.2218234

# Response

**Published:** 2023-10-04

**Authors:** Sufyan Dogra

**Affiliations:** Bradford Institute for Health Research, UK

**Born in Bradford Age of Wonder cohort: A protocol for qualitative longitudinal research – response to reviewer 2** **Reviewer 2’s comment** **Authors’ response** **Overall comments**

- I’m thrilled to see the development of the Age of Wonder cohort, and even more thrilled to see that you are integrating qualitative longitudinal research into this cohort from the start. While the UK is a global leader in cohort study research, the country has not done nearly as well at integrating qualitative research into its major cohorts. I’m very glad to see BiB bucking that trend through this ambitious and important project. 
- It was interesting to read reviewer 1’s thoughtful comments and the authors’ equally thoughtful responses.

Thank you very much for your encouraging and valuable feedback, and critical appreciation.  We really appreciate your attention to detail and identifying areas we had overlooked. Due to our embeddedness in Bradford, we took such information for granted (e.g. Bradford being in the UK). Thanks for highlighting to us the international QLR studies where we can mutually share, learn and align on global similarities and differences of adolescent experiences. We have addressed your main comments throughout the protocol. Thanks for your detailed feedback.

**Abstract**

- It would be helpful for the abstract to provide a bit of information about Bradford. At a minimum I’d suggest mentioning that it is in the UK: this isn’t something that international readers would necessarily know. But ideally you’d also say a little bit more to indicate why Bradford is a particularly interesting site of study. I suspect that including this information in the abstract will help you capture more readers’ interest.

We are very grateful for your observation, and we have added the following sentence at the beginning of the abstract to provide contextual information on inequalities in the UK as well as elaborating on the ethnic and socio-economic status of Bradford. “Bradford is multi-ethnic and one of the most deprived districts in the United Kingdom where around a quarter of children live in low-income families.”

**Introduction**

- Good introduction. The sentence “Forty-five percent of mothers in the cohort are of Pakistani origin and over half live within the fifth most deprived areas of England and Wales” is potentially confusing though. By “fifth” do you mean “one-fifth” i.e. the bottom quintile or do you literally mean the number 5 most deprived area in a ranked list of all UK areas? Logic dictates that it’s the former, but the wording is slightly unclear.

Many thanks for your encouraging words. We agree that the word ‘fifth’ can be confusing, and yes, we do mean to indicate the bottom quintile. To avoid confusion for the reader, we have removed the word in favour of ‘some of the’: “Forty-five percent of mothers in the cohort are of Pakistani origin and over half live within **some of the** most deprived areas of England and Wales.” We believe it this is a simple description of deprivation in relation to ethnicity, which is what we are seeking to capture here.  

- When discussing previous studies undertaking QLR with adolescents, you cite some valuable UK research but may also want to consider international QLR research on adolescence, in particular the mixed methods Young Lives project. There is much that can be learned (empirically and methodologically) from the Young Lives team’s work in countries such as Ethiopia.

     

- In your “Aims” subsection, I was interested to see that you plan to “undertake focused qualitative studies to investigate specific research questions”. Are you able to say how these focused studies fit in with the plans outlined in Table 2? E.g. do you plan on using some of the different approaches outlined in Table 2 to undertake these focused studies?

  Thanks for highlighting our focus on UK research. We now include references to the Young Lives study (Barnett et al., 2013 and Favara et al., 2021). We believe there will be a lot of learning for us from national and international cohort studies, particularly in terms of ethics, recruitment, and research administration. Considering our multi-ethnic, diverse sample, we believe that we too have something unique to share with other longitudinal qualitative cohort studies, both nationally and internationally. We explore avenues for international collaboration in future.  

 Thanks for this very interesting inquiry on how we translate our aims through focused qualitative studies with specific research questions investigating how to improve the health and wellbeing of young people across specific themes. Within BiB we have action research programmes such as the Youth Resilience Programme in Barkerend (one deprived neighbourhood in Bradford). Here we are focused on collecting data from young people from a sub-sample of our QLR cohort on how growing up with positive experiences in early adolescence can prevent young people from violence, drugs, and anti-social behaviour in later adolescence. We have dedicated research team members conducting longitudinal qualitative research on these specific research questions, using creative qualitative methods, such as those outlined in Table 2. We have referred to this in the co-production, involvement and engagement of young people section. We have added two sentences at the end of the penultimate paragraph: “For example, BiB is coordinating an action research programme known as the Youth Resilience Programme in Barkerend (a deprived neighbourhood in Bradford). By working closely with a consortium of small community organisations in Barkerend, we are collecting data from a sub-sample of our QLR cohort on how growing up with positive experiences in early adolescence can prevent young people from violence, drugs, and anti-social behaviour in later adolescence.”  

**Methods**  

- I like your “recruitment in tranches” approach. In addition to the benefits you cite, this recruitment strategy will also enable you as a research team to be a bit more reflective and strategic about recruitment then you might have been if you are recruiting 100 participants in one go. It would also reduce the likelihood of being overwhelmed in Year 1 of data collection.

     

- I’m glad that you will continue to follow individuals who move out of Bradford, and agree with your list of benefits to this approach. There may for example be interesting findings when comparing young people who choose to live at home while attending university versus those who move away for university.

             

- On the notion of consent, universities’ ethics processes often err on the side of bombarding potential participants with information, so that universities are protected but participants are no wiser. I’m thinking for example of overly wordy information sheets that cause participants to think “this is too long to read so I’ll just sign it”. I’m glad to see that you are going to be taking a richer and more holistic approach to informed consent. This is particularly important with regard to issues such as reading level (as mentioned by Reviewer 1).

 

- In Table 1, “Art and culture” appears under “Personal life”. I’m not saying this is the wrong place, but art and culture definitely stands out as somewhat different from more obviously personal issues such as body image. For the category “Social media” you have provided a bit of extra text to suggest how this category might fit into one’s personal life. It might be useful to do the same for “Art and culture”.

         

- I applaud the “citizen scientists” aspect of your project. However you don’t really explain what you mean by this term until the penultimate paragraph of the paper. It would be helpful to bring this explanation forward.

 

- Can you say what you mean by “allow young people to identify pathways which they may not previously have considered”? If this means that you expect your research to potentially shape rather than merely capture your participants’ lives, it would be useful to reflect on this. Such reflections would be very interesting, particularly given the fact that the vast majority of cohort study research posits itself as being as neutral as possible, with regard to influencing study members’ lives (with obvious exceptions such as the discovery of threatening health conditions).

               

- In Table 2, I think your “different Ns for different methods” approach is sensible. If you chose to use all types of approaches on all 100 participants, your project would likely become almost exclusively a data collection and management exercise, rather than an analytic study. (There are some excellent Timescapes-related articles on this phenomenon.) My only real question here is with regard to the “Critical events” category: 5 seems like a small number. Would it be possible to push this number up to 8-10 in order to capture a wider set of experiences of critical events? I’d also be tempted to push the life history number up to 12-15 if possible. But you have to make decisions based on your resources – and in my experience it’s important to be brutally realistic about what can be accomplished in a large qualitative longitudinal study.

 

- You suggest a broad range of data analysis strategies. I wonder if narrative analysis might also be useful – this is a particularly valuable approach for exploring identity development over time. It might also be useful for exploring some of the important issues discussed in paragraph 2 of your Discussion.

   

- In the “Transparency and use of data” section I would expect to see information about what the research team will eventually do with the collected data. For example, ESRC guidelines require UK birth cohort data to be deposited with the UK Data Service so that other researchers can use the data, unless it is impossible up for such data to be sufficiently de-identified. Will your data eventually be made available to the broader research community? If not, it would be helpful to discuss the reasons for keeping it proprietary.

 

- Under “Study status” you refer to September 22 in the future tense. Given the evolving nature of this article, you may want to tweak that.

  Thank you for your kind words. We are glad that we didn’t recruit all 100 young people in Y1. We have found this recruitment in tranches beneficial in Y1, as we have been able to balance the demands of recruitment with the demands of data collection and relationship building vis-à-vis capacity of our QLR research team. Recruiting in tranches has allowed us to reflect and alter our recruitment approach for Y2 and Y3. Moving forwards, we will be recruiting in small clusters from communities that are currently under-represented in our sample (e.g. inner-city males, young people with disabilities, young people vulnerable to exploitation and criminality, etc.).  

 Thanks for your observation on mobility dynamics on young people. We agree that it will be interesting to compare findings when our sample begin to enter higher education and/or the workforce, and the influence this will have on where they choose to live. Many of our sample have already expressed a desire to attend university. It is interesting that nationally the University of Bradford has a significant number of local students, many of whom are from ethnic minority backgrounds. The reasons for students not moving out of the district for university education are economic, cultural, marginalisation, and discrimination. Students who move out of Bradford will provide us with diverse and enriched perspectives on their life choices, economic mobility, etc.    

 Thank you for this thoughtful comment. We are mindful that in a longitudinal study such as this, consent is a fluid and ongoing process. Our team has recently received accessibility training which has provided us with the necessary tools to refine our ethics documents and make them more comprehensible to a wider audience (accounting for lower reading age, etc.). We are advocating for less and less information on consent forms and information sheets because lengthy and bureaucratic consent forms can intimidate people living with marginalisation and disadvantage.    

 Thank you for highlighting this. We realise that we have misplaced ‘art and culture’ under ‘personal life’. Following your thoughtful observation we have placed it in the ‘social and community life’ section, and added ‘civic participation, Bradford as City of Culture for 2025’. This is because our QLR work coincides with the government declaring Bradford as City of Culture for 2025 (BiB data played an instrumental role in Bradford’s selection as City of Culture). Exploring more in the coming years (before and after 2025) on how young people have engaged with everything that Bradford as City of Culture 2025 offers, will provide us with rich insights into how the benefits of cultural provisions are distributed across ethnicity and deprived neighbourhoods.    

 Thanks for your encouragement. We agree it would be helpful to move this explanation earlier. We have moved part of our explanation on citizen science into the ‘co-production, involvement, and engagement of young people’ sub-section of the ‘Methods’ section.  

 Thanks for pointing out what we believe needed more reflection. We understand that we did not elaborate enough on the extent of young peoples’ involvement in a variety of programmes by BiB, linked with our QLR. We do not claim to be neutral researchers but BiB’s hallmark is to shape policy and change lives for better health and wellbeing outcomes. In light of your comment, we have added the following in the QLR sub-section in ‘Methods’: “We acknowledge the potential for our QLR to shape rather than merely observe and describe participants’ lives. Through our longitudinal research and by involvement of young people in other BiB programmes on offering activities around positive experiences of growing up for teenagers, implementation of healthy behaviours, shaping policy and regulations by local authorities (young people’s involvement in research was instrumental in the creation of Bradford’s Clean Air Zone), young people can empower themselves to navigate career pathways and make life choices confidently.”    

 Thanks for your observation and prudent conclusion. We envisage that in future we will have more grants and funding secured that will enable us to increase our sample size across methods. As it stands, the numbers included in Table 2 indicate what is possible with resources available. We acknowledge that small sample size around critical events and life history is a limitation to otherwise promising QLR.                    

 Thank you for highlighting the potential usefulness of narrative analysis for our QLR. We already have collected some data that young people produced by writing poems in small groups, and rap reflecting on their hopes, dreams, and fears for the future. We can see the value in including narrative analysis over the course of our QLR. We have included a reference to Riessman (1993) in our analysis section.    

 Thanks for highlighting this missing information. We are in the process of finding out the best possible way for data to be de-identified and become available to the broader research community. We have started a series of meetings with our IT development and database management teams at BiB to answer the issue of accessibility and best possible ways for our longitudinal data to be used by the broader research community. Once we have a secure system in place we believe our data will be available to the broader research community.       Thank you for highlighting this. We have amended the tense of this section accordingly.  

**iscussion **

- You sensibly suggest that your study will “juxtapose qualitative and quantitative findings, whilst also exploring additional methods of synthesis”. Given the richness of the qualitative and quantitative data that will be available to you, you may want to explore the production of mixed methods pen portraits such as those outlined in Carpentieri, Carter & Jeppesen (2023) ^1^ . Laura Sheard has of course done interesting work on qualitative pen portraits.

 

- I’m pleased to see that you want to make the process fun/enjoyable for participants. Your coproduction strategies will be absolutely essential to achieving that ambition. 

  Thanks for highlighting this, and directing us to your informative paper on the use of pen portraits. We are using creative and artistic methods of collecting qualitative longitudinal data, such as portraits of young people, poetry, music, body-mapping, collage, and youthful activism. We are open to the inclusion of pen portraits and all other creative methods of inquiry.      

 Thank you for your comment. Ensuring participation is enjoyable is a key priority for us and Y1 has proven to us that our participants have a great appetite for participating in data collection, frequently asking us ‘when is the next thing?’. We attribute this to consulting with young people about their hopes for the future of the project. **Miscellaneous **   We have added a reference to Carpentieri et al. (2023) into the QLR sub-section of the Methods section. In Table 2 we have changed the wording of ‘short expression’ to ‘creative expression’ to ensure continuity with our ethics documents, and to represent the emphasis on creativity rather than shortness. This change is echoed throughout the protocol.

# Reviewer report: Reviewer response for version 1

**DOI:** 10.21956/wellcomeopenres.20067.r53552

**Published:** 2022-12-19

**Authors:** Kirstin Mitchell, Raquel Bosó Perez

**Affiliations:** 1 MRC/CSO Social and Public Health Sciences Unit, University of Glasgow, Glasgow, UK

**Overall comments**

 A very interesting proposal from a team who has the skills and expertise to manage the complexity of longitudinal qualitative fieldwork. I really appreciated the participatory and creative approaches the team has proposed.

 The main comments regarding this protocol are:

- To consider the ethical challenges of group research with young people
- To reconsider the approach to sexuality (the only topic section to be limited to 16+) – for more information see comments on Table 1.
- To be more explicit and offer more detail about the participatory nature of the project (participant payment, co-analysis, more detail on what being a citizen scientist entails etc.).
- Consider adding a section on the management of long-term relationships with participants (ethically, practically etc.)
  - Small aside, also worth mentioning given the length of this project - how will you promote continuity/stability in the research team?

 

**Page 3**

**Introduction**

 Paragraph 1 - Was there a longitudinal qualitative component to the BiB study? if so, I think it’s worth flagging in the introduction

**Page 4**

**Qualitative longitudinal research background**

 Paragraph 1 – clear strong rationale for the use of multimodal and creative qualitative methods

**Methods - sampling**

 Paragraph 2 – you mention that the sample will reflect the “overall distribution in the wider BiB cohort.” Does the BiB cohort reflect the distribution of Bradford?

 Paragraph 6 – you use the term “children in special education”, the term is considered offensive by some, might be best to say "children in learning disability education". Would you exclude children with physical disabilities? if not you might want to use the broader term of disabled children (person first language is advocated by disability advocates)

 Paragraph 6 – regarding other underrepresented groups you will include – will you include refugee children (not just children of refugees) and have you considered whether you will include LGBT or questioning adolescents?

**Page 5**

**Methods - sampling**

 Paragraph 1 – you mention “participants to remain a part of our QLR even if they move

 away from Bradford” this is interesting - would be great to provide a rationale for this approach to make clear to the reader the conceptual value of this.

 Paragraph 2 – you talk about how to minimise attrition. Will young people be remunerated for their time and contributions? This would help with attrition rates and the in line with the ethos of equipping them to be citizen scientists

**Consent**

 Overall section comments: I would move the paragraph in "transparency and use of data" (2nd within the section) on the lack of need for reconsent and process for data withdrawal to this section. I found myself writing a lot of questions around reconsent, parental involvement and withdrawal when reading this section that got answered later on when I got to the end of the paper.

 Paragraph 4 – regarding the accessibility of study materials - could also help to check the readability/reading age of all materials produced to ensure they match the average reading age for participants and their parents (check Flesch–Kincaid readability metrics). I have also seen projects focused on including young people under the age of 16 (e.g. Queer Futures 2) use a video with voice over and close captions to increase the accessibility of their study materials - might be worth considering for this project.

 Paragraph 4 – How will you assess Gillick competency? Might be worth developing a series of questions as part of a decisional competency assessment. Asking young people to explain their project in their own words, to explain what they understand the pros and cons to be etc.

 Paragraph 6 – regarding power dynamics involved in parental consent, adolescent assent - if there is space mention explicitly in a couple of lines what these power dynamics are/how they manifest.

**Page 6**

**Table 1**

 Row 1, Personal life – Regarding the subject of *puberty*, the average age for puberty in the UK is 11 for girls and 12 for boys - the topic won’t be relevant to young people if they can only discuss it after the age 16. It feels inconsistent that participants can talk about body image but not how their puberty is shaping this. I would strongly reconsider discussing this topic with younger age groups.

 Has the limit of 16+ been discussed as part of the co-production with young people? It feels on to place a limit on this and no other topic given that sexuality if a normal and health part of adolescent development

 Row 1, Personal life – Regarding the subject of *sexual encounters*, by *encounters* do you mean partnered sex? or will the subject be broader (crushes, sexual fantasies, masturbation, pornography)?  A lot of these subject will be relevant to young people earlier and it is important have an avenue for young people to bring them up earlier. This is a subject that research shows is relevant to young people before the age of 16 and that participants will likely already be discussing in sex education classes, youth groups, community sexual health drop ins etc.

**Qualitative longitudinal research**

 Question for the section - will the research be carried out in a range of languages or only in English? If the former, please talk about how this will impact analysis (e.g., will things be analysed in Urdu or Polish or will transcripts be translated into English and then analysed?)

 Paragraph 1 – I really like the approach of fostering citizen scientist

 Paragraph 2 – Line 4 – “young people and discussions of ethics.” - with ethics boards or about ethics?

 Paragraph 2 – how will people be shortlisted for the in-depth interviews and portrait session?

 Paragraph 3 – Line 5 – “that66” – space missing between words

**Page 7**

**Table 2**

 Rows 7 and 8 – Critical events and life history - will you give young people the option to generate it themselves or will it be created by a researcher (and maybe checked with participants?). Will you give young people the option to generate it themselves or will it be created by a researcher (and maybe checked with participants?)

 Row 10 – WhatsApp Group - legally WhatsApp in the European Region (which includes the UK) cannot be used by under 16 year olds https://faq.whatsapp.com/695318248185629. Furthermore, I have wider concerns about a WhatsApp group for young people with no adult oversight. Do you have plans on how you will deal with inappropriate behaviour within the WhatsApp group? How will you manage potential feelings of exclusion/creation of an in-out group if some participants don’t opt in? This is particularly important to consider as you will have participants who have a learning disability, participants who might not be proficient in English, young people who are vulnerable (and likely to have experiences trauma) as they are from a refugee family or have experience of the care system.

**Reflexivity**

 Question for the section - In practice how will you enact reflexivity? will you keep individual or group diaries, will there be meetings dedicated to reflecting?

 Paragraph 3 – regarding power relations and peer relations, will you use member validation of analysis to aid in equalising this power dynamic?

**Page 8**

**Dissemination and engagement**

 Bullet point 2 – Newsletter - might be worth increasing its frequency - this would help young people feel looped in and might also help minimise attrition rates.

 Bullet point 9 - Girls’ knowledge café - will you run these with any other key groups? e.g., boys cafe, LGBT cafe, a cafe for young people from specific ethnic groups, a cafe for young people with disabilities?

**Transparency and use of data**

 Paragraph 2 - I would mention this (“By virtue of the range of dissemination techniques outlined above, it will be possible to involve young people in certain aspects of data analysis and identifying key information for dissemination.”) in the analysis section - and mention a bit more about how this collaborative analysis will be approached

 Paragraph 3 – move to the consent section

**Ethical approval**

 Paragraph 5 - I think it would be important to mention the specific ethical challenges that can come up in group research (workshops, focus groups, WhatsApp group etc).

**Page 9**

**Ethical approval**

 Paragraph 1 – breach of confidentiality if concerned about safety - how will you define and implement this? This is quite a broad term might be worth using more exact language e.g., in situations of immediate harm to the young person or someone else.

**Discussion**

 Paragraph 4 - this is the first mention of decolonisation - I think it would be useful to spell out how and this this project is decolonising methods.

 Paragraph 6 - Connect to wider literature on group/community/participatory approaches to data generation.

Is the study design appropriate for the research question?

Yes

Is the rationale for, and objectives of, the study clearly described?

Yes

Are sufficient details of the methods provided to allow replication by others?

Partly

Are the datasets clearly presented in a useable and accessible format?

Yes

Reviewer Expertise:

qualitative methods, young people's sexual health and wellbeing

We confirm that we have read this submission and believe that we have an appropriate level of expertise to confirm that it is of an acceptable scientific standard.
